# Loss of NDUFS1 promotes gastric cancer progression by activating the mitochondrial ROS-HIF1α-FBLN5 signaling pathway

**DOI:** 10.1038/s41416-023-02409-5

**Published:** 2023-08-29

**Authors:** Tao Chen, Dongbao Li, Yunliang Wang, Xiaochun Shen, Anqi Dong, Chao Dong, Kaipeng Duan, Jiayu Ren, Weikang Li, Gege Shu, Jiaoyang Yang, Yufeng Xie, Fuliang Qian, Jin Zhou

**Affiliations:** 1 Department of General Surgery, the First Affiliated Hospital of Soochow University, 215006 Suzhou, China; 2 Department of Respiratory Medicine, the First Affiliated Hospital of Soochow University, 215006 Suzhou, China; 3 Department of Thoracic Surgery, the First Affiliated Hospital of Soochow University, 215006 Suzhou, China; 4 Center for Systems Biology, Suzhou Medical College of Soochow University, 215123 Suzhou, China; 5 Medical Center of Soochow University, 215123 Suzhou, China; 6 Jiangsu Province Engineering Research Center of Precision Diagnostics and Therapeutics Development, Soochow University, 215123 Suzhou, China

**Keywords:** Gastric cancer, Gastric cancer

## Abstract

**Background:**

Recent studies suggested that NDUFS1 has an important role in human cancers; however, the effects of NDUFS1 on gastric cancer (GC) are still not fully understood.

**Methods:**

We confirmed that NDUFS1 is downregulated in GC cells through western blot immunohistochemistry and bioinformation analysis. The effect of NDUFS1 on GC was studied by CCK-8, colony formation, transwell assay in vitro and Mouse xenograft assay in vivo. Expression and subcellular localization of NDUFS1 and the content of mitochondrial reactive oxygen species (mROS) was observed by confocal reflectance microscopy.

**Results:**

Reduced expression of NDUFS1 was found in GC tissues and cell lines. Also, NDUFS1 overexpression inhibited GC cell proliferation, migration, and invasion in vitro as well as growth and metastasis in vivo. Mechanistically, NDUFS1 reduction led to the activation of the mROS-hypoxia-inducible factor 1α (HIF1α) signaling pathway. We further clarified that NDUFS1 reduction upregulated the expression of fibulin 5 (FBLN5), a transcriptional target of HIF1α, through activation of mROS-HIF1α signaling in GC cells.

**Conclusions:**

The results of this study indicate that NDUFS1 downregulation promotes GC progression by activating an mROS-HIF1α-FBLN5 signaling pathway.

## Introduction

Gastric cancer (GC) is the fourth most common malignant cancer of the digestive tract and the third leading cause of cancer-related mortality worldwide [1, 2]. In 2012, there were 951,600 new GC cases and 723,100 GC-related deaths [1]. In China, GC is the second and fourth-ranking tumor for morbidity among men and women, and the second-deadliest cancer [3]. Surgery is still considered the most effective treatment approach. Yet, at the time of diagnosis, most GC patients present with advanced-stage cancer [4]. GC metastasis, such as peritoneal metastasis, lymph node metastasis, and distant metastasis, is a key factor for poor prognosis of GC patients [5]. Therefore, it is of great significance to reveal the molecular mechanism promoting the progression of GC.

A mitochondrion is a cellular power plant that generates adenosine triphosphate (ATP) and provides energy for cell’s life activities [6]. It is an important cellular stress sensor that regulates cellular signaling transduction, cell metabolism, and other biological behaviors by producing reactive oxygen species (ROS), and reducing small molecules and other metabolites [6]. Mitochondrial dysfunction is closely related to many diseases, including carcinogenesis and cancer development [6–8]. NADH:ubiquinone oxidoreductase core subunit S1 (NDUFS1) is the largest subunit of the mitochondrial complex I that catalyzes the first step of nicotinamide adenine dinucleotide (NADH) oxidation of the respiratory chain within the mitochondria, having a central role in maintaining the stability and function of the mitochondrial complex I [9]. NDUFS1 absence or mutation can reduce the level and catalytic activity of mitochondrial complex I and break the homeostasis of NADH [9, 10]. Recent studies [11, 12] have shown that the expression of NDUFS1 is downregulated in human lung cancer and renal cell carcinoma, which is closely related to tumor stage, distant metastasis, and poor prognosis. Furthermore, other studies suggested using NDUFS1 as a biomarker for the diagnosis of renal cell carcinoma [12]. Moreover, epigenetic inactivation of NDUFS1 by methylation of CpG islands has also been found to be involved in the malignant transformation of ovarian endometriosis [13]. Accumulating evidence suggests that alteration of NDUFS1 may critically contribute to cancer progression.

We have previously demonstrated that NDUFS1 is downregulated in human GC by isobaric tags for relative and absolute quantification (iTRAQ) analysis [14]. Here we further investigated the role of NDUFS1 in GC progression and its potential molecular mechanism. This is the first study that reported how the downregulation of NDUFS1 promotes GC malignant progression by activating the mROS-HIF1α-FBLN5 signaling pathway.

## Materials and methods

### Lentiviruses

Lentivirus harboring *NDUFS1*, *puromycin N-acetyl-tranferase* (*PAC*), and *green fluorescent protein* (*GFP*) (LV-NDUFS1) and corresponding blank control lentivirus (LV) were purchased from Hanbio (Shanghai, China). Lentivirus harboring *NDUFS1* shRNA, *puromycin N-acetyl-tranferase* (*PAC*), and *GFP* (LV-shNDUFS1) and corresponding control lentivirus harboring control shRNA (LV-shcontrol), as well as lentivirus harboring *FBLN5*, *PAC* and *GFP* (LV-FBLN5) and corresponding blank control LV were supplied by Hanbio (Shanghai, China). Lentivirus harboring *FBLN5* shRNA, *BSD*, and *GFP* (LV-shFBLN5) and corresponding control LV-shcontrol were supplied by Novobio (Shanghai, China).

### Parental and transgenic cell lines

Normal human gastric epithelial cell line GES-1 and a panel of human gastric cancer cell lines, including AGS, HGC-27, KATO3, MKN45, N87, and SNU-1 were supplied by the Cell Bank of Type Culture Collection of the Chinese Academy of Sciences (Shanghai, China) and grown in RPMI-1640 medium (HyClone, Logan, UT, USA) containing 10% fetal bovine serum (FBS) (Gibco, Gaithersburg, MD, USA) and 100 U/ml penicillin-streptomycin antibiotics (Beyotime, Beijing, China) in a humidified atmosphere containing 5% CO_2_/95% air at 37 °C. Transgenic GC cell lines, including MKN45-NDUFS1 (MKN45 overexpressing NDUFS1), MKN45-mock (control for MKN45-NDUFS1), N87-shNDUFS1 (N87 interfering NDUFS1), N87-shcontrol (control for N87-shNDUFS1), MKN45-NDUFS1-FBLN5 (MKN45 overexpressing both NDUFS1 and FBLN5), MKN45-NDUFS1-NC (control for MKN45-NDUFS1-FBLN5), N87-shNDUFS1-shFBLN5 (N87 interfering both NDUFS1 and FBLN5) and N87-shNDUFS1-shNC (control for N87-shNDUFS1-shFBLN5) were generated as previously described [15, 16].

### Human GC samples and TMA

Human GC samples (254 paired tumors and adjacent normal tissues) were harvested from 254 GC surgical patients without neoadjuvant therapy at the Department of General Surgery, the First Affiliated Hospital of Soochow University (Suzhou, Jiangsu, China). The tissues were snap-frozen in liquid nitrogen for subsequent WB analysis. They were fixed in 10% neutral formalin and embedded in paraffin, and then subjected to TMA preparation as described previously [15] for IHC analysis.

The present study was approved by the Ethics Committee of the First Affiliated Hospital of Soochow University.

### Athymic nude mice

Four-week-old male athymic BALB/c nude mice were supplied by SLAC (Shanghai, China). All the animals were housed in an environment with a temperature of 22 ± 1 °C, relative humidity of 50 ± 1%, and a light/dark cycle of 12/12 h. All animal studies (including the mice euthanasia procedure) were done in compliance with the regulations and guidelines of Soochow University institutional animal care and conducted according to the AAALAC and the IACUC guidelines.

### Antibodies

Rabbit anti-NDUFS1 (cat. no. ab157221) and mouse anti-FBLN5 (cat. no. ab66339) were supplied by Abcam (Cambridge, MA, USA). Rabbit anti-HIF1α (cat. no. YT2133), anti-GLUT1 (cat. no. YT1928), anti-VEGFA (cat. no. YT5108) and anti-glyceraldehyde-3-phosphate dehydrogenase (GAPDH) (cat. no. YM3215) were supplied by ImmunoWay (Plano, TX, USA). Rabbit anti-Histone H3 (cat. no. 9717) was obtained from CST (Danvers, MA, USA). Horseradish peroxidase (HRP)-conjugated goat anti-rabbit (cat. no. G1213) or anti-mouse (cat. no. G1214) IgG was supplied by Servicebio (Wuhan, Hubei, China). Rabbit anti-HSP60 (cat. no. 15282-1-AP) and mouse anti-TOM20 (cat. no. 66777-1-Ig) were purchased from ProteinTech Group (Chicago, IL, USA). FITC-labeled goat anti-rabbit IgG (cat. no. A0562) and Cy3-labeled goat anti-mouse IgG (cat. no. A0521) were from Beyotime (Shanghai, China).

### Cell biological behavior in vitro and in vivo

CCK-8 assay (Beyotime), colony formation assay, and transwell assay were used to analyze the difference in cell proliferation, colony formation, and cell migration between MKN45-NDUFS1 vs. MKN45-mock and N87-shNDUFS1 vs. N87-shcontrol cells, while Mouse xenograft assay was used to assess cell growth and migration in vivo.

#### CCK-8 assay

CCK-8 assay was performed as previously described [15].

#### Colony formation assay

Cells were plated in six-well plates (200 cells/2 ml medium/well) for 2 weeks. Cells were then fixed with 4% paraformaldehyde (Solarbio, Beijing, China) and stained with 0.5% crystal violet solution (Solarbio). The tumor colonies were counted, and an analysis of the clonogenic ability was subsequently performed.

#### Transwell assay

Transwell assay was performed using an 8-μm-pore size 24-well transwell chamber (Corning, NY, USA); invasion and migration were assessed as previously described [15, 16].

#### Mouse xenograft assay

Cells were subcutaneously injected into nude mice (2 × 10^6^ cells per mouse, five mice per group). Tumor size was evaluated by the formula (tumor volume=0.52 × length × width^2^). Mice were sacrificed at the end of the experiment, and tumors were dissected and weighed. The tumors were then fixed in 10% neutral formalin and embedded in paraffin for following IHC analysis.

To establish a tumor lung metastasis model, the above tumor cells (2 × 10^6^ cells per mouse, five mice per group) were intravenously injected into the tail veins of nude mice. Six weeks after intravenous injection, the mice were euthanized to collect lung tissues for hemoatoxylin & esosin (HE) analysis of lung metastasis nodules.

### IHC analysis

IHC analysis of NDUFS1 (rabbit anti-NDUFS1: 1:100; HRP-conjugated goat anti-rabbit IgG: 1:200) and FBLN5 (mouse anti-FBLN5: 1:500; HRP-conjugated goat anti-mouse IgG: 1:200) in human GC TMA and xenograft tumor tissues was conducted as previously described [15]. Their expression was then assessed by a weighted IHC score, and a weighted score of ≥4 (++ or +++) was considered as a high expression [15].

### Detection of mROS

The level of mROS was detected by MitoSOX^TM^ Red mitochondrial superoxide indicator (Thermo, Waltham, MA, USA) following company’s protocols.

### WB analysis

Proteins extracted from GC tissues and cell lines were subjected to WB analysis of NDUFS1, FBLN5, HIF1α, GLUT1, VEGFA, GAPDH (loading control for total protein) or Histone H3 (loading control for nuclear protein), or HSP60 (loading control for mitochondrial protein) as described previously [15, 16]. The primary antibody anti-NDUFS1 (1:5000), anti-FBLN5 (1:2000), anti-HIF1α (1:1000), anti-GLUT1 (1:1000), anti-VEGFA (1:1000), anti-GAPDH (1:5000), anti-Histone H3 (1:2000) or anti-HSP60 (1:10,000) were used according to company’s instructions. Accordingly, the secondary antibody HRP-conjugated goat anti-rabbit or anti-mouse IgG (1:5000) was used. Band densities were quantified and normalized to GAPDH or Histone H3 loading control.

### RT-qPCR analysis

The total RNA was prepared for SYBR Green-based qPCR analysis of GLUT1, VEGFA, and FBLN5 mRNA levels as previously described [16].

### Confocal reflectance microscopy

After incubation of GC cell lines, NDUFS1 was stained using rabbit anti-NDUFS1 (1:200) and FITC-labeled goat anti-rabbit IgG (1:500), and TOM20 was stained using mouse anti-TOM20 (1:200) and Cy3-labeled goat anti-mouse IgG (1:500) according to the manufacturer’s instructions. After the final washing step with phosphate-buffered saline, the nuclei were counterstained with DAPI (4’,6-diamidino-2-phenylindole) and then visualized the microparticles by CRM (FV3000, Olympus, Japan).

### LUC reporter assay

For LUC reporter analysis of HIF1α transcriptional activity, MKN45-NDUFS1 vs MKN45-mock and N87-shNDUFS1 vs. N87-shcontrol cells were cotransfected with HIF1α-LUC (Firefly) (Genomeditech, Shanghai, China) and pGMLR-TK-LUC (Renilla) (Genomeditech) (internal control) reporter plasmids. Forty-eight hours after transfection, LUC activity was detected using Dual-LUC Assay System (Promega, Madison, WI, USA) according to manufacturer’s instructions. For LUC reporter analysis of FBLN5 promoter activity, wt or mut fragment of human FBLN5 promoter (TSS upstream: from −2000 bp to −1 bp) was inserted into a pGL3-basic vector (Promega) at the upstream of *Firefly LUC* reporter gene to generate pGL3-wt FBLN5 promoter-LUC or pGL3-mut FBLN5 promoter-LUC (negative control) reporter plasmid. The constructs and internal control pGMLR-TK-LUC were then used for LUC reporter analysis of FBLN5 promoter activity in GC cells, as mentioned above.

### mROS inhibitor assay

N87-shNDUFS1 cells were pretreated for 10 μM Mito-TEMPO (APExBIO, Houston, TX, USA) or DMSO (vehicle control) for 1 h and then subjected to mROS inhibitor functional assay and expression analysis of HIF1α, GLUT1, VEGFA, and FBLN5.

### HIF1α knockdown assay

N87-shNDUFS1 cells were transfected with 50 nM HIF1α siRNA (siHIF1α) (CST) or control siRNA (sicontrol) (CST) for 24 hours and then subjected to HIF1α knockdown functional assay and expression analysis of HIF1α and FBLN5.

### FBLN5 rescue and knockdown assays

MKN45-NDUFS1-FBLN5 and N87-shNDUFS1-shFBLN5 cells were subjected to FBLN5 rescue and knockdown functional assays, respectively.

### Statistical analysis

IHC scoring data were presented as −, +, ++ or +++. Categorical data of high or low expression were presented as a percentage of total cases. Measurement data of functional and mechanistic studies were analyzed by a normal distribution test and then presented as mean ± standard deviation (SD) when *P* > 0.1. Before ANOVA was carried out, measurement data were further analyzed by the homogeneity of variance test (*P* > 0.1 indicates homogeneity of variance). All statistical tests, including Mann–Whitney *U* test, Pearson’s *χ*^2^ test, Log-rank test, Spearman rank correlation coefficient, Student’s *t* test, and analysis of variance (ANOVA) with least significant difference (LSD) post hoc multiple comparisons were performed with SPSS20.0 (SPSS, Chicago, IL, USA). A two-sided value of *P* < 0.05 indicated a statistically significant difference.

## Results

### NDUFS1 protein is decreased in GC

**Fig. 1:**
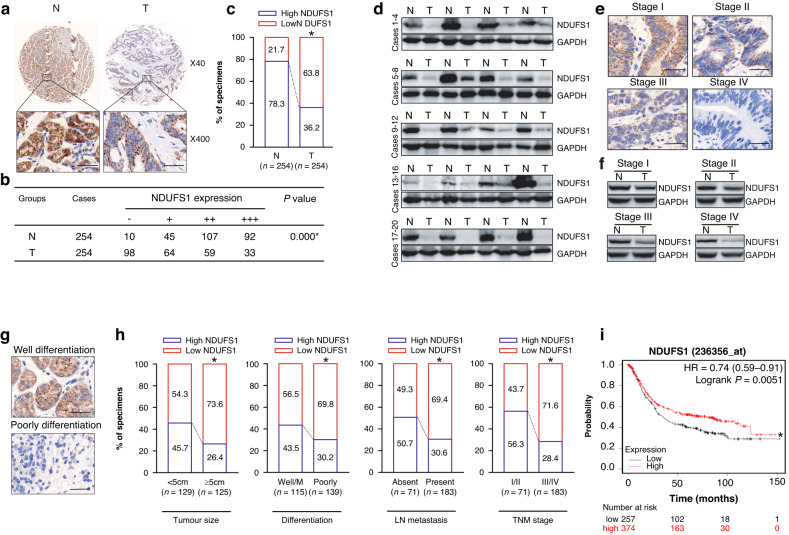
NDUFS1 expression is reduced in GC clinical tumor tissues and its association with clinical malignant features and survival of GC. **a** Representative NDUFS1 IHC images of GC tumor tissues and matched adjacent normal tissues. Scale bars: 100 μm. **b** NDUFS1 IHC scoring in 254 paired GC tumor tissues and matched adjacent normal tissues. *P < 0.05, Mann–Whitney *U* test. **c** Percentage of high (“++” and “+++”) and low (“−” and “+”) NDUFS1 expression between GC tumor tissues and matched adjacent normal tissues. *P < 0.05, Pearson’s *χ*^2^ test. **d** Representative NDUFS1 WB images of GC tumor tissues and matched adjacent normal tissues. **e** Representative NDUFS1 IHC images of TNM Stage I–IV GC tumor tissues. Scale bars: 100 μm. **f** Representative NDUFS1 WB images of I–IV GC tumor tissues and matched adjacent normal tissues. N matched adjacent normal tissues, T tumor tissues. **g** Representative NDUFS1 IHC images of well/poorly differentiated GC tumor tissues. Scale bars: 100 μm. **h** Association of NDUFS1 expression level with tumor size, differentiation, lymph node (LN) metastasis, and TNM stage of GC. *P < 0.05, Pearson’s *χ*^2^ test. **i** Association of NDUFS1 expression level with the survival of GC from the GEO database by Kaplan–Meier Plotter (https://kmplot.com/analysis/). The cut-off value for this analysis is 379. *P < 0.05, Log-rank test.

Our previous iTRAQ analysis [14] has suggested that the expression of NDUFS1 is downregulated in clinical GC tumor tissues. To confirm these findings, we further analyzed the NDUFS1 expression in GC by immunohistochemistry (IHC) analysis using tissue microarray (TMA) that included 254 paired GC tumor tissues and adjacent normal gastric tissues. As shown in Fig. 1a–c, NDUFS1 expression was abundant in normal tissues but decreased in GC tissues (*P* < 0.05), which is consistent with our iTRAQ data [14].

Next, we used Western blot (WB) to detect NDUFS1 in snap-frozen GC and adjacent normal gastric tissues from the same specimens. WB analysis (Fig. 1d) showed that the NDUFS1 was reduced in GC tissues compared with the adjacent normal gastric tissues.

Interestingly, we found that the expression level of NDUFS1 was weak in Stage I/II GC tumors and barely detectable in Stage III/IV GC tumors (Fig. 1e, f), indicating that the expression of NDUFS1 gradually decreased as GC progresses to more advanced stages. Additionally, NDUFS1 expression was lower in poorly differentiated GC tumors than that in well differentiated GC tumors (Fig. 1g).

### Downregulation of NDUFS1 is associated with malignant features and poor prognosis

To investigate the association of NDUFS1 with clinicopathological features of GC, 162 GC patients with low NDUFS1 expression (− and +) were classified as the NDUFS1-low expression group, and 92 GC patients with high NDUFS1 expression (++ and +++) were classified as the NDUFS1-high expression group. The correlation of NDUFS1 with clinicopathological variables of GC, such as gender and age of patients, as well as tumor size, differentiation, lymph node metastasis, and Tumor Node Metastasis (TNM) stage, was then evaluated. Our data (Table 1 and Fig. 1h) showed that a low level of NDUFS1 was correlated with large tumor size, poor differentiation, presence of lymph node metastasis, and high TNM stage (*P* < 0.05). Moreover, the survival curve (Fig. 1i) which was analyzed from the Gene Expression Omnibus (GEO) database by Kaplan–Meier Plotter demonstrated that low NDUFS1 expression was associated with the poor prognosis of GC patients (*P* < 0.05). Therefore, our data, together with GEO data, suggested that the expression level of NDUFS1 was inversely correlated with malignant features but positively correlated with good prognosis in GC, implying that the decrease of NDUFS1 may greatly contribute to GC progression.

**Table 1:** Relationship of NDUFS1 expression level with GC clinicopathological features.

Variables	High NDUFS1 (*n* = 92)	Low NDUFS1 (*n* = 162)	*P* value
Sex			
Male	65	109	0.579
Female	27	53	
Age (years)			
<60	18	29	0.743
≥60	74	133	
Tumor size (cm)			
<5	59	70	0.001*
≥5	33	92	
Tumor differentiation			
Well and moderately differentiated	50	65	0.029*
Poorly differentiated	42	97	
Lymph node metastasis			
Absent	36	35	0.003*
Present	56	127	
TNM stage			
Early (I/II)	40	31	<0.001*
Late (III/IV)	52	131	

^*^P < 0.05, Pearson’s *χ*^2^ test.

### NDUFS1 is mainly located in the mitochondria

**Fig. 2:**
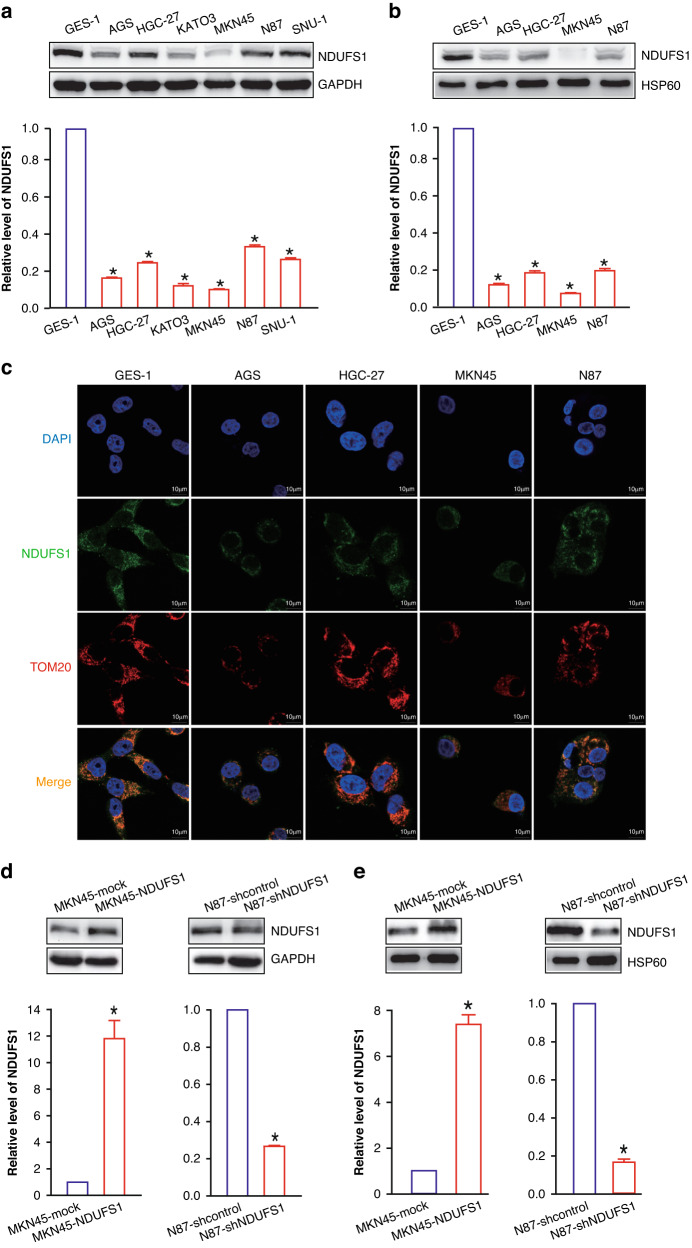
The expression of NDUFS1 is reduced in GC cell lines and is mainly located in mitochondria. **a** WB analysis of NDUFS1 in GC cell lines and normal gastric epithelial cell lines. Representative WB images (upper) and relative protein level (lower) of NDUFS1 in GC cell lines (GES-1 normal gastric epithelial cell line served as a control; with 1 being the value for GES-1) were shown. *P < 0.05, ANOVA with LSD post hoc multiple comparisons, *n* = 6 per group. **b** WB analysis of NDUFS1 in mitochondria of GC cell lines and normal gastric epithelial cell line. Representative WB images (upper) and relative protein level (lower) of NDUFS1 in GC cell lines (GES-1 normal gastric epithelial cell line served as a control; with 1 being the value for GES-1) were shown. *P < 0.05, ANOVA with LSD post hoc multiple comparisons, *n* = 6 per group. **c** Representative images of immunofluorescence colocalization of NDUFS1 (green) and TOM20 (red) in GC cell lines and normal gastric epithelial cell lines. Scale bars = 10 μm. **d** WB analysis of the overexpression or knockdown efficiency of NDUFS1 in GC cells. Representative WB images (upper) and relative protein level (lower) of NDUFS1 in MKN45-NDUFS1 (MKN45-mock served as a control; with 1 being the value for MKN45-mock) and N87-shNDUFS1 (N87-shcontrol served as a control; with 1 being the value for N87-shcontrol) GC cells were shown. *P < 0.05, Student’s *t* test, *n* = 6 per group. **e** WB analysis of NDUFS1 in mitochondria of above cells. Representative WB images (upper) and relative protein level (lower) of NDUFS1 in MKN45-NDUFS1 (MKN45-mock served as a control; with 1 being the value for MKN45-mock) and N87-shNDUFS1 (N87-shcontrol served as a control; with 1 being the value for N87-shcontrol) GC cells were shown. *P < 0.05, Student’s *t* test, *n* = 6 per group.

To assess the expression of NDUFS1 in GC cells, the AGS, HGC-27, KATO3, MKN45, N87, and SNU-1 human GC cell lines as well as the GES-1 normal human gastric epithelial cell line were used. As shown in (Fig. 2a), all GC cell lines expressed a lower level of NDUFS1 when compared with the GES-1 control cell line (*P* < 0.05). In accordance with clinical tissue data, our cell model data also demonstrated that NDUFS1 expression was downregulated in GC.

To further determine the subcellular localization of NDUFS1, mitochondria of AGS, HGC-27, MKN45, and N87 were extracted using a mitochondrial isolation kit (Thermo Scientific, 89874). Moreover, the expression level of NDUFS1 in mitochondria of the above four GC cell strains (Fig. 2b) was detected by WB, and the trend was consistent with the results in Fig. 2a. Based on the above results, we further observed the expression and subcellular localization of NDUFS1 in gastric cancer cell lines using confocal fluorescence microscopy (Fig. 2c). Compared with GES-1 the expression level of NDUFS1 in AGS and MKN45 was lower, while the expression level of NDUFS1 in HGC-27 and N87 was higher. However, in both GES-1 and AGS, HGC-27, MKN45, and N87, NDUFS1 (green fluorescence) basically overlapped with mitochondrial protein TOM20 (red fluorescence), indicating that NDUFS1 was mainly localized in mitochondria.

Next, NDUFS1 overexpression was achieved by infecting MKN45 and AGS GC cells with lentivirus carrying full-length human *NDUFS1* coding sequence (CDS), whereas its knockdown was achieved by infecting N87 and HGC-27 GC cells with lentivirus carrying short hairpin RNA (shRNA) targeting *NDUFS1* (*P* < 0.05) (Fig. 2d and Supplementary Fig. S1A). The levels of NDUFS1 protein in mitochondria of NDUFS1-overexpressing MKN45 GC cells and NDUFS1-interfering N87 GC cells were detected by WB analysis (Fig. 2e), and the changing trend was consistent with expectation.

### NDUFS1 inhibits the proliferation, migration, invasion, and metastasis of GC in vitro and in vivo

**Fig. 3:**
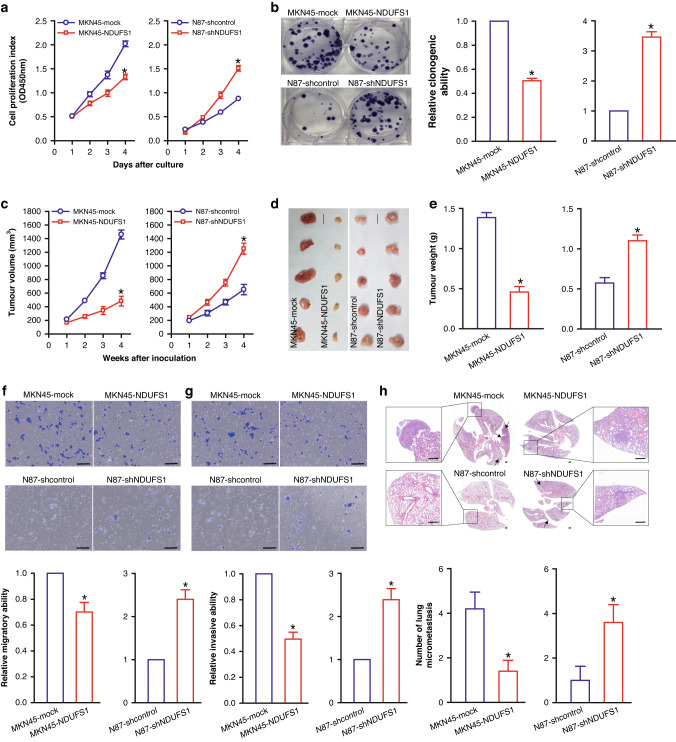
NDUFS1 inhibits the proliferation and metastasis of GC cells in vitro and in vivo in athymic nude mice. **a** CCK-8 cell proliferation assay. *P < 0.05, Student’s *t* test, *n* = 6 per group. **b** Colony formation assay. Representative tumor colony images (left) and relative clonogenic ability (right) of MKN45-NDUFS1 (MKN45-mock served as a control; with 1 being the value for MKN45-mock) and N87-shNDUFS1 (N87-shcontrol served as a control; with 1 being the value for N87-shcontrol) GC cells were shown. *P < 0.05, Student’s *t* test, *n* = 6 per group. **c** Volume of GC xenograft tumors. *P < 0.05, Student’s *t* test, *n* = 5 per group. **d** Images of GC xenograft tumors. Scale bars: 1 cm. **e** Weight of GC xenograft tumors. *P < 0.05, Student’s *t* test, *n* = 5 per group. **f** Transwell migration assay. Representative images of transwell migration assay (upper) and relative migratory ability (lower) of MKN45-NDUFS1 **(**MKN45-mock served as a control; with 1 being the value for MKN45-mock) and N87-shNDUFS1 (N87-shcontrol served as a control; with 1 being the value for N87-shcontrol) GC cells were shown. Scale bars: 100 μm. *P < 0.05, Student’s *t* test, *n* = 6 per group. **g** Transwell invasion assay. Representative images of transwell invasion assay (upper) and relative invasive ability (lower) of MKN45-NDUFS1 (MKN45-mock served as a control; with 1 being the value for MKN45-mock) and N87-shNDUFS1 (N87-shcontrol served as a control; with 1 being the value for N87-shcontrol) GC cells were shown. Scale bars: 100 μm. *P < 0.05, Student’s *t* test, *n* = 6 per group. **h** HE analysis of tumor lung metastasis colonies. Representative HE images (upper) and the number of tumor metastasis colonies (lower) were shown. Tumor lung metastasis colonies in HE images were marked with arrows. Scale bars: 200 μm. *P < 0.05, Student’s *t* test, *n* = 5 per group.

Using the above-mentioned NDUFS1-overexpressing or -interfering GC cell model, we performed cell counting kit-8 (CCK-8) and colony formation assays in vitro and tumor xenograft assay in vivo in athymic nude mice. Compared with the MKN45 and AGS-mock control cells, overexpression of NDUFS1 inhibited the proliferation ability and clonogenicity in MKN45 and AGS GC cells (all *P* < 0.05) (Fig. 3a, b and Supplementary Fig. S1B, C). By contrast, the knockdown of NDUFS1 promoted these capabilities in N87 and HGC-27 GC cells compared with the N87 and HGC-27-shcontrol control cells (all *P* < 0.05) (Fig. 3a, b). Moreover, in vivo data further showed that overexpression of NDUFS1 suppressed MKN45 xenograft tumor growth (*P* < 0.05), whereas knockdown of NDUFS1 accelerated N87 xenograft tumor growth (*P* < 0.05) (Fig. 3c–e). Our results demonstrated that NDUFS1 was a key negative regulator of GC proliferation and growth.

To examine whether NDUFS1 affects the metastasis ability of GC cells, we analyzed the effect of NDUFS1 overexpression or knockdown on GC cell migration and invasion in vitro and lung metastasis in vivo in athymic BALB/c nude mice using NDUFS1-overexpressing MKN45/AGS and NDUFS1-interfering N87/HGC-27 GC cells. In vitro transwell migration (Fig. 3f and Supplementary Fig. S1E) and invasion (Fig. 3g and Supplementary Fig. S1F) assays showed that overexpression of NDUFS1 suppressed the migration and invasion potential of MKN45 and AGS GC cells when compared to the MKN45 and AGS-mock control cells (*P* < 0.05). Conversely, compared to the N87 and HGC-27-shcontrol control cells, knockdown of NDUFS1 enhanced the migration and invasion potential in N87 and HGC-27 GC cells (*P* < 0.05) (Fig. 3f, g and Supplementary Fig. S1E, F).

In vivo lung metastasis assay (Fig. 3h) in athymic nude mice further demonstrated that NDUFS1 overexpression impaired the in vivo distant lung metastasis ability of MKN45 GC cells (*P* < 0.05), whereas its knockdown enhanced this ability of N87 GC cells (*P* < 0.05). Our data showed that NDUFS1 could attenuate the metastasis potential of GC cells, indicating that NDUFS1 may potentially function as a metastasis inhibitor in GC.

### NDUFS1 impedes GC progression via downregulating the mROS-HIF1α signaling pathway

**Fig. 4:**
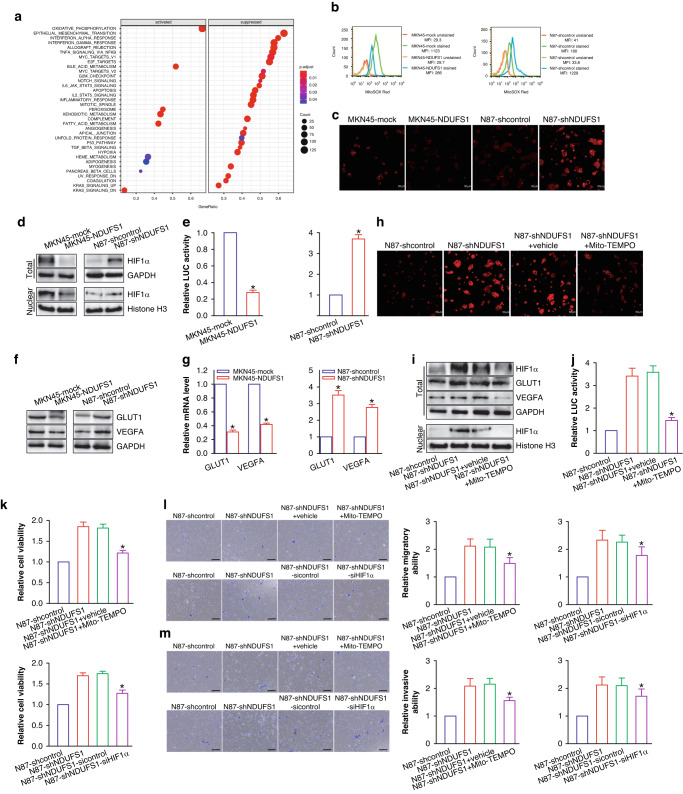
NDUFS1 attenuates the progression of GC by inactivating the mROS-HIF1α signaling pathway. **a** GSEA gene enrichment analysis of NDUFS1 in GC from TCGA database by R language. **b** Representative images of flow cytometric analysis of mROS in GC cells. MFI, mean fluorescent intensity. **c** The level of ROS detected by fluorescence microscopy. Scale bars = 50 μm. **d** WB analysis of HIF1α in GC cells. Representative WB images (left) and relative protein level (right) of total or nuclear HIF1α in MKN45-NDUFS1 (MKN45-mock served as a control; with 1 being the value for MKN45-mock) and N87-shNDUFS1 (N87-shcontrol served as a control; with 1 being the value for N87-shcontrol) GC cells were shown. total or nuclear HIF1α: *P < 0.05, Student’s *t* test, *n* = 6 per group. **e** LUC reporter gene analysis of HIF1α’s transcriptional activity. Relative LUC activity of MKN45-NDUFS1 (MKN45-mock served as a control; with 1 being the value for MKN45-mock) and N87-shNDUFS1 (N87-shcontrol served as a control; with 1 being the value for N87-shcontrol) GC cells was shown. *P < 0.05, Student’s *t* test, *n* = 6 per group. **f** WB analysis of GLUT1 and VEGFA in GC cells. Representative WB images (upper) and relative protein level (lower) of GLUT1 and VEGFA in MKN45-NDUFS1 (MKN45-mock served as a control; with 1 being the value for MKN45-mock) and N87-shNDUFS1 (N87-shcontrol served as a control; with 1 being the value for N87-shcontrol) GC cells were shown. GLUT1 or VEGFA: *P < 0.05, Student’s *t* test, *n* = 6 per group. **g** RT-qPCR analysis of GLUT1 and VEGFA in GC cells. Relative mRNA level of GLUT1 and VEGFA in MKN45-NDUFS1 (MKN45-mock served as a control; with 1 being the value for MKN45-mock) and N87-shNDUFS1 (N87-shcontrol served as a control; with 1 being the value for N87-shcontrol) GC cells was shown. GLUT1 or VEGFA: *P < 0.05, Student’s *t* test, *n* = 6 per group. **h** The level of ROS de*t*ected by fluorescence microscopy. Scale bars = 50 μm. **i** Representative images of WB analysis of HIF1α and its targets GLUT1 and VEGFA after inhibition of mROS. **j** LUC reporter gene analysis of HIF1α’s transcriptional activity after inhibition of mROS. Relative LUC activity was shown, with 1 being the value for N87-shcontrol. *P < 0.05, N87-shNDUFS1+Mito-TEMPO compared with N87-shNDUFS1+vehicle control, ANOVA with LSD post hoc multiple comparisons, *n* = 6 per group. **k** CCK-8 assay after inhibition of mROS or knockdown of HIF1α in N87-shNDUFS1 GC cells. Relative cell viability was shown, with 1 being the value for N87-shcontrol. **l** Transwell migration assay after inhibition of mROS or knockdown of HIF1α in N87-shNDUFS1 GC cells. Relative migratory ability was shown, with 1 being the value for N87-shcontrol. **m** Transwell invasion assay after inhibition of mROS or knockdown of HIF1α in N87-shNDUFS1 GC cells. Representative images of transwell invasion assay (left) and relative invasive ability (right) were shown, with 1 being the value for N87-shcontrol. Scale bars: 100 μm. **k**–**m**: *P < 0.05, N87-shNDUFS1+Mito-TEMPO compared with N87-shNDUFS1+vehicle control; or N87-shNDUFS1-siHIF1α compared with N87-shNDUFS1-sicontrol control, ANOVA with LSD post hoc multiple comparisons, *n* = 6 per group.

In order to analyze the signaling pathways that NDUFS1 may be involved in, we divided all GC tissues into high expression group and a low expression group according to the expression level of NDUFS1 in the TCGA database and then performed GSEA gene enrichment analysis (Fig. 4a) according to the expression of all genes in these two groups by using R language. It has been reported that NDUFS1 downregulation may attenuate oxygen consumption and enhance the level of mROS in neurons [17]. It has also been shown that mROS can stabilize HIF1α protein and activate HIF1α signaling, leading to the tumor’s malignant progression [6, 8, 18]. To delineate the mechanism through which NDUFS1 modulates GC progression, we investigated whether the downregulation of NDUFS1 may induce the production of mROS and activate the mROS-HIF1α signaling pathway in GC cells. Our data (Fig. 4b) demonstrated that the level of mROS was significantly decreased in NDUFS1-overexpressing MKN45 GC cells, whereas the mROS level was increased in NDUFS1-interfering N87 GC cells, compared with the respective control cells.

To further verify the levels of mROS in NDUFS1-overexpressing MKN45 GC cells and NDUFS1-interfering N87 GC cells, immunofluorescence was used to analyze the content of mROS; the results are shown in Fig. 4c. Furthermore, overexpression of NDUFS1 markedly downregulated the protein levels of total and nuclear HIF1α in MKN45 cells, while knockdown of NDUFS1 upregulated total and nuclear HIF1α in N87 cells (*P* < 0.05) (Fig. 4d). Luciferase (LUC) reporter assay for detection of transcriptional activity also showed that NDUFS1 inhibited the transcriptional activity of HIF1α in GC cells (*P* < 0.05) (Fig. 4e). Consistently, NDUFS1 suppressed the expression of classical HIF1α transcriptional targets such as glucose transporter type 1 (GLUT1) and vascular endothelial growth factor A (VEGFA) at both the mRNA and protein levels in GC cells (*P* < 0.05) (Fig. 4f, g). Our data revealed that the expression reduction of NDUFS1 enhanced the level of mROS as well as the expression level and transcriptional activity of HIF1α in GC cells.

To examine whether NDUFS1 regulated HIF1α signaling via mROS, we performed an mROS inhibitor assay in NDUFS1-interfering N87 GC cells using a mitochondria-targeted antioxidant Mito-TEMPO (Fig. 4h). As we expected, inhibition of mROS remarkably blunted the NDUFS1 knockdown-elicited upregulation of HIF1α’s expression (Fig. 4i) and transcriptional activity (Fig. 4i, j) in N87 cells (*P* < 0.05), further demonstrating that NDUFS1 downregulation promoted the activation of HIF1α signaling by inducing the production of mROS in GC cells. More importantly, mROS inhibitor and HIF1α small interfering RNA (siRNA) functional assays (Fig. 4k–m) showed that not only inhibition of mROS but also siRNA-mediated knockdown of HIF1α abrogated NDUFS1 knockdown-elicited growth and metastasis in N87 cells (*P* < 0.05). Collectively, our results clarified that NDUFS1 could inhibit GC progression by suppressing the mROS-HIF1α signaling pathway. Conversely, the reduction of NDUFS1 may facilitate GC progression via activating the mROS-HIF1α signaling pathway.

### NDUFS1 downregulates FBLN5 of GC cells via inhibiting the mROS-HIF1α signaling pathway

**Fig. 5:**
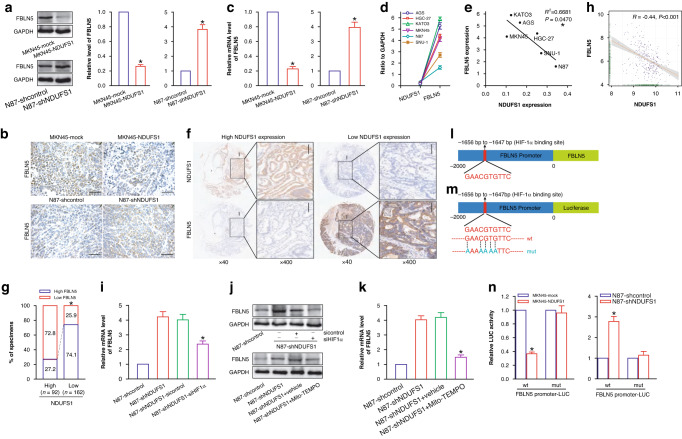
NDUFS1 inhibits the expression of FBLN5 via impairing mROS-HIF1α signaling in GC cells. **a** WB analysis of FBLN5 in GC cells. Representative WB images (left) and relative protein level (right) of FBLN5 in MKN45-NDUFS1 (MKN45-mock served as a control; with 1 being the value for MKN45-mock) and N87-shNDUFS1 (N87-shcontrol served as a control; with 1 being the value for N87-shcontrol) GC cells were shown. *P < 0.05, Student’s *t* test, *n* = 6 per group. **b** Representative FBLN5 IHC images of GC xenograft tumor tissues. Scale bars: 50 μm. **c** RT-qPCR analysis of FBLN5 in GC cells. Relative mRNA level of FBLN5 in MKN45-NDUFS1 (MKN45-mock served as a control; with 1 being the value for MKN45-mock) and N87-shNDUFS1 (N87-shcontrol served as a control; with 1 being the value for N87-shcontrol) GC cells was shown. *P < 0.05, Student’s *t* test, *n* = 6 per group. **d** WB analysis of expression of endogenous NDUFS1 (NDUFS1/GAPDH) and FBLN5 (FBLN5/GAPDH) in GC cells. **e** Endogenous expression relationship between NDUFS1 and FBLN5 in GC cells. *P < 0.05, Spearman rank correlation coefficient. **f** Representative NDUFS1 and FBLN5 IHC images of GC clinical tumor tissues. Scale bars: 100 μm. **g** Percentage of GC tumor tissue specimens showing high or low NDUFS1 expression in relation to the expression level of FBLN5. *P < 0.05, Pearson’s *χ*^2^ test. **h** Pearson correlation analysis of NDUFS1 and FBLN5 expression using GEO database GSE62254. *P < 0.05, Pearson’s *χ*^2^ test. **i** RT-qPCR analysis of FBLN5 after knockdown of HIF1α. Relative mRNA level of FBLN5 was shown, with 1 being the value for N87-shcontrol. *P < 0.05, N87-shNDUFS1-siHIF1α compared with N87-shNDUFS1-sicontrol control, ANOVA with LSD post hoc multiple comparisons, *n* = 6 per group. **j** Representative images of WB analysis of FBLN5 after knockdown of HIF1α or inhibition of mROS. **k** RT-qPCR analysis of FBLN5 after inhibition of mROS. Relative mRNA level of FBLN5 was shown, with 1 being the value for N87-shcontrol. *P < 0.05, N87-shNDUFS1+Mito-TEMPO compared with N87-shNDUFS1+vehicle control, ANOVA with LSD post hoc multiple comparisons, *n* = 6 per group. **l** Binding site of HIF1α and FBLN5 promoter predicted by JASPAR database. **m** Construction sketch map of LUC reporter gene vector of wt and mut FBLN5 promoter. **n** LUC reporter gene analysis of the activity of FBLN5 promoter. Relative LUC activity in MKN45-NDUFS1 (MKN45-mock served as a control; with 1 being the value for MKN45-mock) and N87-shNDUFS1 (N87-shcontrol served as a control; with 1 being the value for N87-shcontrol) GC cells was shown. wt FBLN5 promoter: *P < 0.05, Student’s *t* test, *n* = 6 per group.

FBLN5 is highly expressed and closely related to malignant clinicopathological factors, including poor differentiation, lymph node metastasis, and high tumor stage in GC [19], indicating FBLN5 exhibits tumor-promoting effects in GC. Furthermore, we have demonstrated that GC tissues display a low level of NDUFS1 as well as a high level of FBLN5 by iTRAQ analysis [14]. These cues led us to hypothesize that the inverse expression correlation of NDUFS1 and FBLN5 would occur in GC and that NDUFS1 would suppress GC progression by downregulating the expression of FBLN5. Therefore, we preliminarily analyzed the regulatory effect of NDUFS1 on FBLN5 in NDUFS1-overexpressing and -interfering GC cells and their tumor xenografts. WB (Fig. 5a) and IHC (Fig. 5b) analyses showed that overexpression of NDUFS1 inhibited the expression of FBLN5 in MKN45 GC cells in vitro and in vivo in athymic nude mice (*P* < 0.05), whereas knockdown of NDUFS1 enhances the expression of FBLN5 in N87 GC cells and xenografts (*P* < 0.05). RT-qPCR (Fig. 5c) analysis also demonstrated that NDUFS1 downregulated the mRNA expression level of FBLN5 in GC cells (*P* < 0.05). Additionally, the endogenous expression level of NDUFS1 showed a negative association with FBLN5 in both GC cell lines (*P* < 0.05) (Fig. 5d, e) and clinical GC tissues (*P* < 0.05) (Fig. 5f, g). Moreover, bioinformatics analysis of correlation between NDUFS1 and FBLN5 expression verified the above conclusion (*P* < 0.05) (Fig. 5h). Our results revealed that NDUFS1 negatively modulated the expression of FBLN5 in GC.

Guadall et al. [20] have demonstrated that hypoxia can upregulate the expression of FBLN5, a transcriptional target of HIF1α, in endothelial cells through activation of HIF1α signaling. To address whether NDUFS1 suppresses the FBLN5 expression via attenuating mROS-HIF1α signaling in GC cells, we conducted HIF1α siRNA and mROS inhibitor assays in NDUFS1-interfering N87 GC cells and subsequently analyzed FBLN5 by RT-qPCR and WB, respectively. We found that the knockdown of HIF1α impaired the NDUFS1 knockdown-induced upregulation of FBLN5 in NDUFS1-interfering N87 GC cells (*P* < 0.05) (Fig. 5i, j). Moreover, inhibition of mROS, accompanied by downregulation of HIF1α, also produced a similar inhibitory effect on the expression of FBLN5 (*P* < 0.05) (Fig. 5j, k).

We further predicted the potential binding sites of the HIF1α transcription factor and FBLN5 promoter by JASPAR database analysis. As shown in Fig. 5l, there was a HIF1α-binding sequence at −1656 bp to −1647 bp (GAACGTGTTC) of FBLN5 transcription start site (TSS) upstream in the FBLN5 promoter, in which ACGTG was completely consistent with the hypoxia response element (HRE) core sequence A/GCGTG [21]. We then constructed pGL3-wild type (wt) FBLN5 Promoter-LUC and pGL3-mutant (mut) FBLN5 Promoter-LUC (used as a negative control) vectors (Fig. 5m) to carry out LUC reporter assay (Fig. 5n) in NDUFS1-overexpressing and -interfering GC cells. Furthermore, we found that overexpression of NDUFS1 downregulated the LUC activity in pGL3-wt FBLN5 Promoter-LUC-transfected MKN45 GC cells (*P* < 0.05), while knockdown of NDUFS1 exerted an opposing regulatory effect in pGL3-wt FBLN5 Promoter-LUC-transfected N87 GC cells (*P* < 0.05), confirming that FBLN5 is indeed a transcriptional target of HIF1α. To the best of our knowledge, this is the first study that elucidated the molecular mechanisms where NDUFS1 transcriptionally suppressed the expression of FBLN5 in GC cells via inactivating HIF1α signaling through downregulating mROS.

### FBLN5 is critically involved in NDUFS1 loss-induced GC progression

**Fig. 6:**
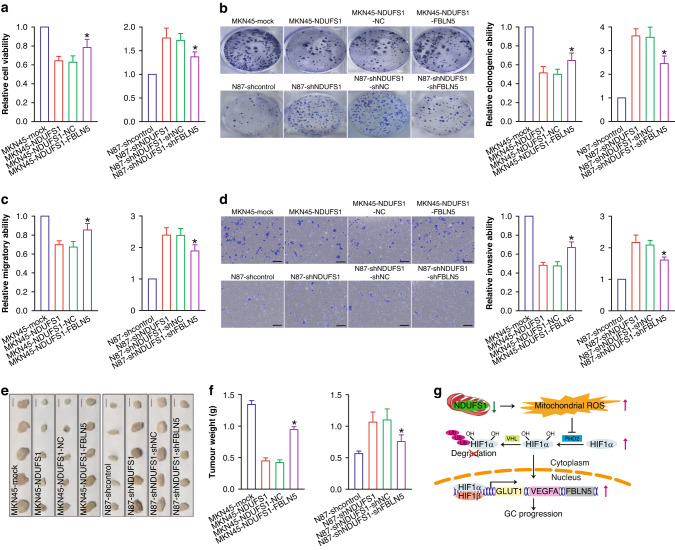
FBLN5 is responsible for NDUFS1 loss-elicited GC progression. **a** CCK-8 assay of FBLN5 rescue in MKN45-NDUFS1 or knockdown in N87-shNDUFS1. Relative cell viability was shown, with 1 being the value for MKN45-mock and N87-shcontrol, respectively. **b** Colony formation assay of FBLN5 rescue in MKN45-NDUFS1 or knockdown in N87-shNDUFS1. Representative tumor colony images (left) and relative clonogenic ability (right) were shown, with 1 being the value for MKN45-mock and N87-shcontrol, respectively. **c** Transwell migration assay of FBLN5 rescue in MKN45-NDUFS1 or knockdown in N87-shNDUFS1. Relative migratory ability was shown, with 1 being the value for MKN45-mock and N87-shcontrol, respectively. **d** Transwell invasion assay of FBLN5 rescue in MKN45-NDUFS1 or knockdown in N87-shNDUFS1. Representative images of transwell invasion assay (left) and relative invasive ability (right) were shown, with 1 being the value for MKN45-mock and N87-shcontrol, respectively. Scale bars: 100 μm. **a**–**d**: *P < 0.05, MKN45-NDUFS1-FBLN5 compared with MKN45-NDUFS1-NC control; or N87-shNDUFS1-shFBLN5 compared with N87-shNDUFS1-shNC control, ANOVA with LSD post hoc multiple comparisons, *n* = 6 per group. **e** Images of GC xenograft tumors in tumor xenograft assay of FBLN5 rescue in MKN45-NDUFS1 or knockdown in N87-shNDUFS1. Scale bars: 1 cm. **f** Weight of GC xenograft tumors in FBLN5 rescue or knockdown tumor xenograft assay. *P < 0.05, MKN45-NDUFS1-FBLN5 compared with MKN45-NDUFS1-NC control; or N87-shNDUFS1-shFBLN5 compared with N87-shNDUFS1-shNC control, ANOVA with LSD post hoc multiple comparisons, *n* = 5 per group. **g** A schematic model of NDUFS1’s function during GC progression.

To address the role of FBLN5 in the NDUFS1 loss-elicited GC progression, we performed the FBLN5-knockdown and -overexpression functional assays, respectively. The knockdown of FBLN5 impaired the growth and metastasis in N87-shNDUFS1 GC cells (*P* < 0.05), whereas overexpression of FBLN5 rescued the growth and metastasis in MKN45-NDUFS1 GC cells (*P* < 0.05) (Fig. 6a–f). Our data demonstrated that FBLN5 as an important mediator, was crucial in NDUFS1 loss-induced GC progression. In summary, our study provided the first evidence (Fig. 6g) that the loss of NDUFS1 expression in GC cells was conducive to the activation of mROS-HIF1α signaling pathway and the induction of transcriptional expression of HIF1α downstream genes such as GLUT1, VEGFA, and FBLN5, which are responsible for the malignant progression of GC.

## Discussion

Dysfunction of mitochondrion contributes to carcinogenesis and cancer development [6, 8, 18, 22, 23]. Therefore, elucidating the roles and mechanisms of mitochondrial abnormalities in cancer cells may help to develop novel and effective cancer therapeutics. The mitochondrial respiratory chain is composed of complexes I, II, III, and IV, transmitting hydrogen and electrons to the ATP synthase complex to synthesize energy and maintain the transmembrane hydrogen ion gradient cycle [24]. Mitochondrial complex I consist of 45 subunits (14 core subunits and 31 accessory subunits) [9, 23, 25]. Recent reports [11, 12] have shown that NDUFS1 expression is very low in lung cancer and renal cell carcinoma. In this study, we further confirmed our previous unbiased iTRAQ analysis [14]. Furthermore, GC cells have been reported to exhibit a deficiency of mitochondrial complex I and decreased respiratory capacity [26], which to a certain extent, supports our finding.

Mitochondrial DNA mutations participate in mitochondrial dysfunction and tumor progression in GC [27, 28]. However, the effect of the defect of mitochondrial complex I components, such as NDUFS1, in GC progression remained unclear. Our biological assays demonstrated that decreased NDUFS1 accelerated GC cell proliferation and metastasis in vitro and in vivo in athymic nude mice, indicating that mitochondrial complex I subunit NDUFS1 was a novel tumor suppressor. Of note, inhibition of mitochondrial complex I activity by reduction of core subunit NDUFS3 [29] or NDUFV1 [30] as well as accessory subunit NUDFB9 [31] has been found to enhance the aggressive potential of breast cancer. The observed phenomena is consistent with our conclusion that the downregulation of NDUFS1 promotes GC progression.

ROS, mainly derived from the mitochondrion, is the oxygen-free radical molecule produced during oxidative stress [7, 18]. The mitochondrial complex I performs the first step of oxidative phosphorylation and is the main contributor to the production of mROS [9, 25]. An abnormal mitochondrial respiratory chain can trigger the increase of mROS that induces the oxidative damage of lipids, proteins, and nucleic acids, resulting in genomic instability and carcinogenesis [6–8, 18]. Moreover, mROS can stimulate cancer cell proliferation, antiapoptosis, metastasis, and metabolic adaptation via multiple mechanisms, leading to cancer progression [6–8, 18]. For example, mROS has been shown to promote the activation of HIF1α signaling via stabilizing the HIF1α protein, which is required for cancer progression [6, 8, 18, 32, 33]. Interestingly, the defect of NDUFS1 can impair the level and catalytic activity of mitochondrial complex I [9, 10], thereby enhancing mROS production in neurons [17]. Therefore, we assumed that the downregulation of NDUFS1 would promote GC progression by activating the mROS-HIF1α signaling pathway. In support of our hypothesis, we analyzed the effect of NDUFS1 on the level of mROS as well as the expression and transcriptional activity of HIF1α in GC cells. As expected, the reduction of NDUFS1 facilitated the production of mROS and the accumulation and nuclear translocation of HIF1α in GC cells. Accordingly, the reduction of NDUFS1 enhanced the transcriptional activity of HIF1α and upregulated the expression of HIF1α downstream genes such as GLUT1 and VEGFA [32, 33]. These data revealed that NDUFS1 reduction promotes GC progression by activating the mROS-HIF1α signaling pathway.

HIF1α, a major regulatory factor that promotes angiogenesis, is abnormally high expressed in a variety of tumors and is closely related to tumor metastasis [34]. Currently, the main targets of antiangiogenesis drugs used in treatment of tumors are focused on VEGF-VEGFR pathway [34]. Ramucirumab, the only second-line antiangiogenesis drug approved by the FDA for the treatment of advanced GC, is a monoclonal antibody that binds to VEGFR2 and prevents its activation [35]. This study demonstrated that downregulation of NDUFS1 in GC cells can activate HIF1α signaling pathway and thus upregulate VEGFA expression. Therefore, it is worth studying the clinical correlation of NDUFS1 expression level with tumor angiogenesis as well as clinical efficacy of Ramucirumab in GC patients. Perhaps NDUFS1 can be used as a predictive biomarker for sensitivity to Ramucirumab in GC patients.

FBLN5 as a member of the fibulin family of extracellular matrix proteins is an important elastic fibrin that is widely distributed in tissues rich in elastic fibers [36, 37]. FBLN5 is involved in elastic fiber formation, vascular development, wound repair and cell adhesion, migration, and proliferation [36, 37]. FBLN5 mutation or downregulation causes skin relaxation, vascular sclerosis, emphysema, and other senile diseases [38]. The abnormality of FBLN5 is also closely related to the occurrence and development of cancer [39, 40]. FBLN5 is downregulated in a majority of cancer types [39, 40], such as liver cancer [41] and lung cancer [42], displaying cancer-suppressing effects. On the contrary, FBLN5 increases DNA synthesis and stimulates motility in fibrosarcoma cells [43]. FBLN5 promotes epithelial-mesenchymal transition (EMT) in breast cancer cells [44] and mediates Nogo-B-stimulated EMT in cervical cancer cells [45]. Upregulation of FBLN5 enhances GC cell proliferation and invasion in GC, acting as a key factor in GC progression [19]. It has been shown that FBLN5 is a transcriptional target of HIF1α and hypoxia stimulates FBLN5 expression by a HIF1α-dependent mechanism [20]. In addition, hypoxia and transforming growth factor β (TGFβ) can cooperatively promote FBLN5 expression [46]. NDUFS1 downregulation stimulated the expression of FBLN5 of GC cells by activating HIF1α signaling through upregulating mROS. Importantly, FBLN5-knockdown and -functional overexpression assays further verified that FBLN5 was a critical mediator for NDUFS1 loss-elicited promoting effects in GC.

A high level of ROS beyond the tolerance threshold of cancer cells can cause cancer cell death [47]. Mouse double minute 2 (MDM2) can bind to and sequester NDUFS1 to reduce mitochondrial respiration and increase mROS, ultimately leading to apoptosis in lung cancer cells [48]. While supporting our finding that NDUFS1 reduction-induced mROS production in GC, it challenged our finding that NDUFS1 reduction-induced mROS promoted GC progression. A possible explanation for the discrepancy could be different cellular models and approaches used. Different cells may show different sensitivity to ROS-induced oxidative damage. It is worth mentioning that FBLN5 can block ROS production and oxidative damage elicited by fibronectin-β1 integrin signaling in pancreatic cancer via competing with fibronectin for β1 integrin binding [49, 50]. In our NDUFS1 lowly expressed GC model, it remains unclear whether FBLN5 upregulation regulated by mROS-HIF1α signaling is an additional adaptive antioxidant pathway to balance ROS levels and consequently prevent ROS-mediated oxidative stress, which future studies should address.

Overall, our data revealed that the downregulation of mitochondrial complex I subunit NDUFS1 promotes GC progression by activating an mROS-HIF1α-FBLN5 signaling pathway. FBLN5 is a vital mediator for NDUFS1 reduction-elicited GC progression. NDUFS1 may be a novel tumor suppressor and a potential therapeutic target for GC.

### Supplementary information

Fig. S1 legend

Supplementary figure 1

## Data Availability

The data that support the findings of this study are openly available in GEO database at https://www.ncbi.nlm.nih.gov/geo/.
