# Supplementary material for: Loss of NDUFS1 promotes gastric cancer progression by activating the mitochondrial ROS-HIF1α-FBLN5 signaling pathway
Source: Br J Cancer. 2023 Aug 29;129(8):1261–73. doi: 10.1038/s41416-023-02409-5 (PMC10575981; doi:10.1038/s41416-023-02409-5)
Supplement: Supplementary file 1 — Fig. S1 legend [file 41416_2023_2409_MOESM1_ESM.doc]

**Fig.S1 NDUFS1 inhibits the proliferation and metastasis of GC cells *in vitro*. A** WB analysis of the overexpression or knockdown efficiency of NDUFS1 in GC cells. Representative WB images (*upper*) and relative protein level (*lower*) of NDUFS1 in AGS-NDUFS1 (AGS-mock served as a control; with 1 being the value for AGS-mock) and HGC-27-shNDUFS1 (HGC-27-shcontrol served as a control; with 1 being the value for HGC-27-shcontrol) GC cells were shown. *, *P*<0.05, Student’s *t* test, n=6 per group. **B** CCK-8 cell proliferation assay. *, *P*<0.05, Student’s *t* test, n=6 per group. **C** Colony formation assay. *, *P*<0.05, Student’s *t* test, n=6 per group. **D** Wound healing assay. *, *P*<0.05, Student’s *t* test, n=6 per group. **E** Transwell migration assay. Scale bars: 100 μm. *, *P*<0.05, Student’s *t* test, n=6 per group. **F** Transwell invasion assay. Scale bars: 100 μm. *, *P*<0.05, Student’s *t* test, n=6 per group.
