# Supplementary figures and images for: Loss of NDUFS1 promotes gastric cancer progression by activating the mitochondrial ROS-HIF1α-FBLN5 signaling pathway
Source: Br J Cancer. 2023 Aug 29;129(8):1261–73. doi: 10.1038/s41416-023-02409-5 (PMC10575981; doi:10.1038/s41416-023-02409-5)

**Figure S1**

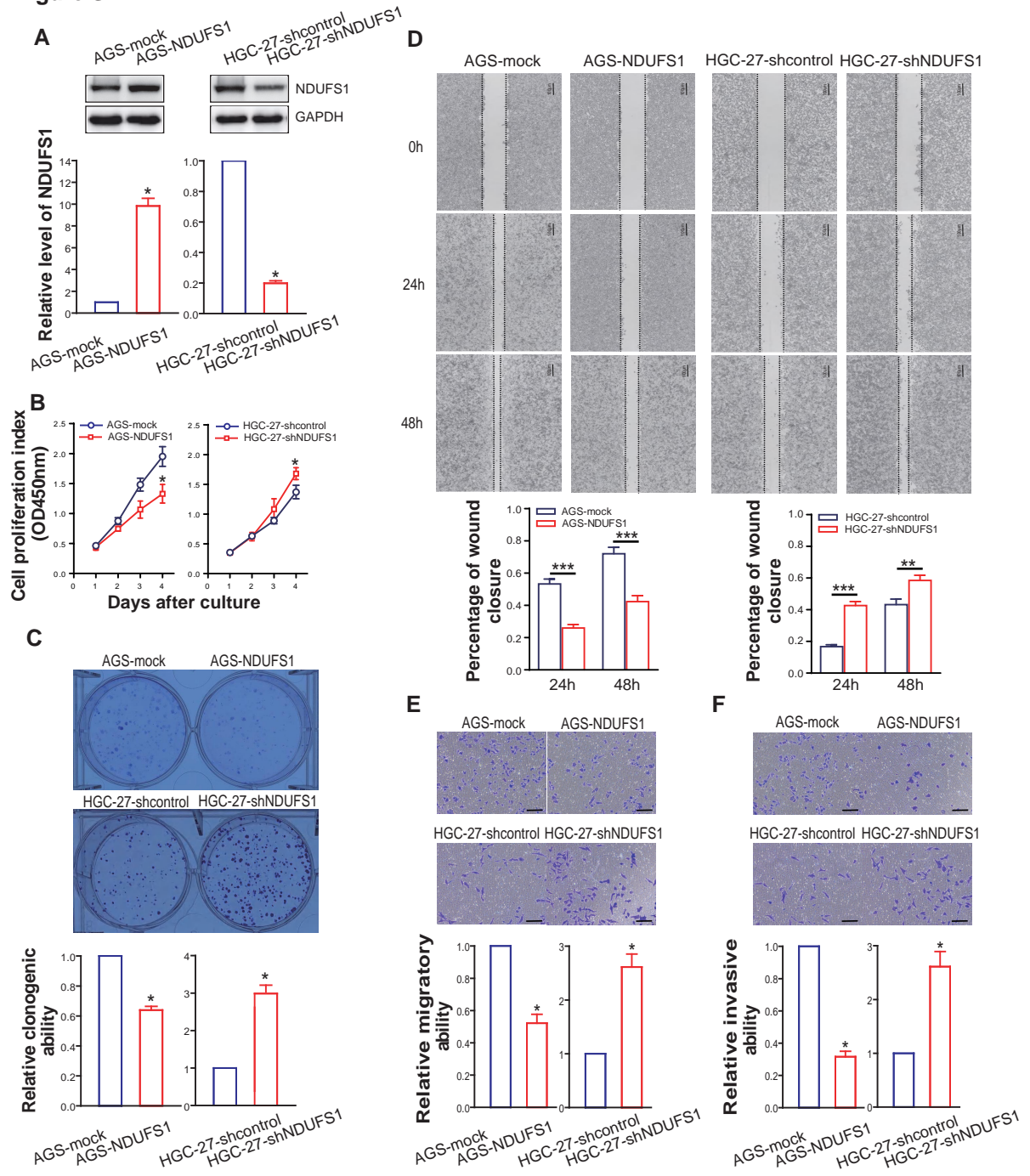

Supplement: Supplementary file 2 — Supplementary figure 1 [file 41416_2023_2409_MOESM2_ESM.pdf]
